# Supplementary material for: Nowcasting the 2022 mpox outbreak in England
Source: PLoS Comput Biol. 2023 Sep 18;19(9):e1011463. doi: 10.1371/journal.pcbi.1011463 (PMC10538717; doi:10.1371/journal.pcbi.1011463)
Supplement: S1 Fig — The grey bars indicate the data available at the time of the nowcast, the white bars are the complete data, and the lines are the nowcasting projection, with 95% prediction intervals in the ribbon. Each panel shows a different lead time, where the nowcasting model is trying to predict that many days prior to the current date. (PDF) [file pcbi.1011463.s001.pdf]

Supplementary Figures

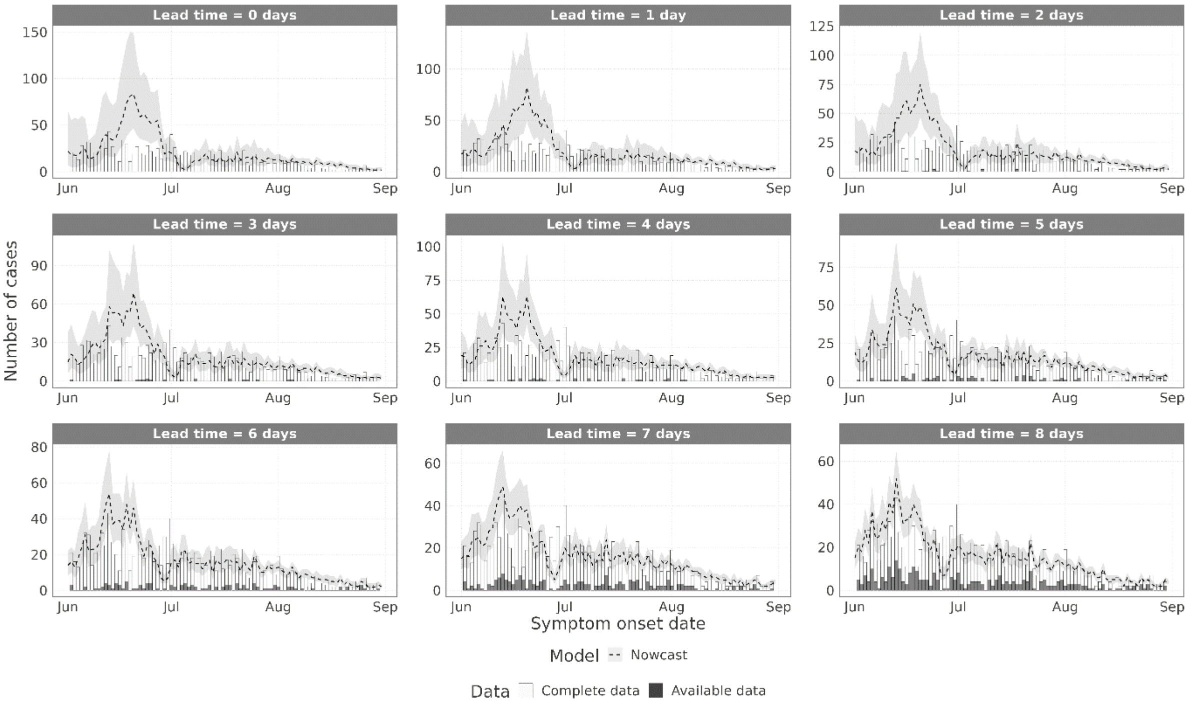

Figure S1: *Performance of the non-parametric and parametric nowcasting models by symptom onset date – full study period. The grey bars indicate the data available at the time of the nowcast, the white bars are the complete data, and the lines are the nowcasting projection, with 95% prediction intervals in the ribbon. Each panel shows a different lead time, where the nowcasting model is trying to predict that many days prior to the current date.*
