# Supplementary material for: Nowcasting the 2022 mpox outbreak in England
Source: PLoS Comput Biol. 2023 Sep 18;19(9):e1011463. doi: 10.1371/journal.pcbi.1011463 (PMC10538717; doi:10.1371/journal.pcbi.1011463)
Supplement: Attachment — Submitted filename: Review_comments_Mpox_Nowcasting_PLOS.pdf [file pcbi.1011463.s002.pdf]

# Review comments

Nowcasting the 2022 mpox outbreak in England

March 2023

## Overall comments

In this well-written manuscript (PCOMPBIOL-D-23-00266) by Overton et al. a nowcasting model using generalised additive models is propsed to correct the 2022 mpox epidemic curve in England. They propose multiple models implemented in an R package and compare their performance. They also highlight the challenges of specific reporting structures (and their possible changes over time) which I find important to note when performing this type of nowcasting. I enjoyed reading the paper but would appreciate some added technical details (specified in major comments). Also, the figures would benefit from a bit more attention (specifeied in minor comments).

## Major comments

- Abstract and line 45. I would appreciate a clearer explanation of what the actual novelty of this model is.
- Line 46. What are the similarities and what is different from the models used by the German Nowcasting Hub (1)?
- Line 223. "Generally do agree" but do they here? I believe a Bayesian version of this model could be fast as well so this argument is rather weak. It also does not align with the former statement about the preference of a non-Bayesian model as it could not be run on the "same IT systems used to access the data" (on line 42).
- Line 394 and 395. Stronger how and to what extent?

## Minor comments

- All figures. That the shaded areas represents is not stated.
- Figure 2. It is rather hard to see the nowcast result in the right panel. Consider shortening the range of dates shown.

- Figure 5. Axis label overlap.
- Figure 6 and 7. Wrong date on the right panel title.
- To visualise the performance over time a suggestion is to also plot the bias or interval score over time to see if stays stable over time or highlight periods of low performance.
- Some plot axis labels are very small making it har to read and the ledgen titel is often strangely named ”Legdend”.

## Referenses

1. German Nowcasting Hub. Nowcasts der COVID-19 Hospitalisierungsinzidenz. https://covid19nowcasthub.de/
