# Supplementary material for: Nowcasting the 2022 mpox outbreak in England
Source: PLoS Comput Biol. 2023 Sep 18;19(9):e1011463. doi: 10.1371/journal.pcbi.1011463 (PMC10538717; doi:10.1371/journal.pcbi.1011463)
Supplement: Attachment — Submitted filename: reviewer_response.docx [file pcbi.1011463.s003.docx]

Dear Editor

We thank the reviewers for their time in considering this paper and their helpful comments. We have addressed the majority of the comments, with detailed responses provided below. For any unaddressed comments we have provided a response saying why not. We have also gone through the entire paper and made small corrections/improvements throughout.

Kind regards

Christopher Overton

**Reviewer #1**

**Major**

- Abstract and line 45. I would appreciate a clearer explanation of what the actual novelty of this model is.
- Changed to: “Therefore, we developed a nowcasting model that makes novel use of individual-level patient data using hierarchical generalised additive models”
- Line 46. What are the similarities and what is different from the models used by the German Nowcasting Hub (1)?
- Changed to: “These models have a similar structure to the epidemic nowcasting models used by German COVID-19 nowcasting hub (13), but we consider both parametric and non-parametric approaches to the reporting delay distribution, making optimal use of the available individual-level patient data.” We have also added more details to the methods sections.
  - Non-parametric model: “The resulting model, with splines used for both the epidemic curve term (s_1 (o)) and reporting delay term (s_2 (d)) is a simplified version of the model described in (19), with penalised cubic regression splines used instead of P-splines. However, instead of extrapolating the reporting delays outside of the observation window, we truncate using the maximum possible reporting delay, as described in Section 3.2.”
  - Parametric model: “This model extends on existing GAM-based nowcasting models (19,24) through the explicit incorporation of the right-truncation corrected reporting delay distribution, f_θ (d), instead of fitting the reporting delay within the nowcasting model. This allows the model to make optimal use of individual-level data describing the reporting process.”
- Line 223. ”Generally do agree” but do they here? I believe a Bayesian version of this model could be fast as well so this argument is rather weak. It also does not align with the former statement about the preference of a non-Bayesian model as it could not be run on the ”same IT systems used to access the data” (on line 42).
- Thanks for highlighting this, and we realise the paragraph did not quite convey our intended meaning. We have changed this to: “To fit this distribution, we use maximum likelihood estimation. We only consider the central estimate for the distribution, rather than propagating distribution uncertainty through the nowcasting model. Extensions using a Bayesian version of this method, such as (22), could be applied to correctly propagate the distribution uncertainty, for example by bootstrapping the nowcasting model across posterior samples of the reporting delay. However, such methods were not applicable since Bayesian packages could not be used on the IT systems used to access the data. Additionally, the model is found to be well calibrated using only the central estimate, so the additional uncertainty would not substantially improve performance.”
- Line 394 and 395. Stronger how and to what extent?
- Changed to: “For both parametric models, the 60 days data range outperforms the 30 days model on all scores. For both non-parametric models, the 30 days data range is optimal. For non-parametric symptom onset date, both data lengths have equal coverage, with reduced bias in the 30 days model. The 60 days model has better interval score, but we are selecting for reduced bias over interval score. For non-parametric specimen date, the 30 days model outperforms the 60 days model on 2 out of 3 scores.”

**Minor**

- All figures. That the shaded areas represents is not stated.
- Figure 2. It is rather hard to see the nowcast result in the right panel.
- Consider shortening the range of dates shown.
- Figure 5. Axis label overlap.
- Figure 6 and 7. Wrong date on the right panel title.
- To visualise the performance over time a suggestion is to also plot the bias or interval score over time to see if stays stable over time or highlight periods of low performance.
- Some plot axis labels are very small making it har to read and the ledgen titel is often strangely named ”Legdend”.

We thank the reviewer for highlighting these issues and have revised all the figures.

Referenses

1. German Nowcasting Hub. Nowcasts der COVID-19 Hospitalisierungsinzidenz. https://covid19nowcasthub.de/

**Reviewer #2: The article investigates a Nowcasting the 2022 mpox outbreak in England**

1Please add the main findings and objective of the current study in the abstract.

Changed to: “We have developed a nowcasting model using generalised additive models that makes novel use of individual-level patient data to correct the mpox epidemic curve in England. The aim of this model is to correct for backfilling in the epidemic curve and provide real-time characteristics of the state of the epidemic, including the real-time growth rate. This model benefited from close collaboration with individuals involved in collecting and processing the data, enabling temporal changes in the reporting structure to be built into the model, which improved the robustness of the nowcasts generated. The resulting model accurately captured the true shape of the epidemic curve in real time.”
- What are the benchmark cases in your Nowcasting the 2022 mpox outbreak in Englandstudy?

The benchmark case is the epidemic curve without correcting for backfilling, which leads to substantial underestimation of the growth rate. E.g. Figure 3.
- What are the special cases of your study?

The special case of this study is the 2022 mpox outbreak in England.
- Table needs to be referenced.

All tables are now referenced in the text.
- Main equations and propositions need to be referenced.

No references are required for the equations in the paper.

- Punctuation is missing after some equations.

We have checked through the article but could not find missing punctuation after any equations.
- Try to show more of the physical situation in the results and discussion to reflect the proposed notion of the wholestudy.

Figures 2, 3 and 4 show the physical situation of the outbreak in England for the three key variables considered in this study. In the discussion, we include this paragraph on the physical situation: “The proposed models were used weekly throughout the mpox outbreak in England, facilitating reliable estimation of timely growth rate estimates, to understand how the outbreak is evolving (14). From the analysis, we can see the clear difference in the epidemic curve across the different data streams. Data on symptom onset date shows the epidemic curve peaking earliest. However, this exact timing of the peak based on symptom onset date is unreliable, since the ascertainment rate of this data was declining at the same time as the peak. Specimen date on the other hand has been reliably recorded throughout the epidemic, leading to a reliably timed peak at the end of June. Similarly, date of report is consistently recorded, allowing reliable timing of the peak, but is further lagged, occurring 1-2 weeks later than the specimen date peak. This demonstrates the value of nowcasting methods, since they can detect the epidemic peak earlier”.

- For enhancing the introduction section with the new publications, old references may be replaced with new ones such as:
<https://doi.org/10.1140/epjp/s13360-023-03865-x>
<https://doi.org/10.1007/s40808-022-01607-z>
<https://doi.org/10.1088/1402-4896/ac7ebc>
<https://doi.org/10.1007/s40808-021-01313-2>

We thank the reviewer for sharing these papers. However, upon review we do not feel any would contribute to the introduction section.

**Reviewer #3: Title: Nowcasting the 2022 mpox outbreak in England**

Author: Christopher E. Overton et al.

Recommendation:

In the submitted manuscript, the authors studied a Nowcasting the 2022 mpox outbreak in England. In my opinion, the results presented in this paper are interesting and I suggest that the paper will be accepted for publication in the journal if the authors can make some revisions according to the following comments.


1- Authors can add the following references to enrich the introductory section: Mathematical analysis of spread and control of the novel corona virus (COVID-19) in China." Chaos, Solitons and Fractals 141 (2020): 110286.
On Analysis of fractional order mathematical model of Hepatitis B using Atangana–Baleanu Caputo (ABC) derivative." Fractals (2021): 2240017.
Caputo type fractional operator applied to Hepatitis B system." Fractals (2021):
The stochastic bifurcation analysis and stochastic delayed optimal control for epidemic model with general incidence function." Chaos: An Interdisciplinary Journal of Nonlinear Science - AIP (2021): 104649.
We thank the reviewer for sharing these interesting papers. However, we do not feel that they add to the introduction of the paper, so have opted not to include them in this paper.

2- The English writing of the paper is required to be improved. Please check the manuscript carefully for typos and grammatical errors. Also, the English structure of the article, including punctuation, semicolon, and other structures, must be carefully reviewed.

We have reviewed the language throughout the paper and made changes where required.

3. They should double check the mathematical formulations, and also add appropriate references for governing equations.

We have fixed the mathematical formulations. No references are required for the equations in the paper.
