# Supplementary material for: Nowcasting the 2022 mpox outbreak in England
Source: PLoS Comput Biol. 2023 Sep 18;19(9):e1011463. doi: 10.1371/journal.pcbi.1011463 (PMC10538717; doi:10.1371/journal.pcbi.1011463)
Supplement: Attachment — Submitted filename: revisions_2.docx [file pcbi.1011463.s004.docx]

The Authors have addressed all the criticisms by all Reviewers. However I have some remaining comments:
1. In Tables 2-4, could the authors explain why interval score for the non-parametric model can be that high (in the order of 1E17)?
2. Could that be dominated by one or two extreme values which may have dominated the assessment?

We thank the reviewer for highlighting these points. As they suspect, the high interval scores are due to a few points where the model estimate shoots off to crazy high numbers which are highly unrealistic. In the original draft, we left these in to demonstrate the instability of the model. Based on this comment, we have adjusted our approach, excluding values where the prediction is over 100 times larger than the true value, which yields more sensible interval scores. We have added the following text to explain and justify this change: “To generate model scores, predictions where the predicted value was over 100 times larger than the true value have been omitted (and the true value is not equal to zero), since these cause the interval score to grow to very large values. This only affects the non-parametric models, which appear to have some occasional numerical instability that leads to very large and unrealistic predictions. Removing these does not affect the validity of the scoring, since in practice such unrealistic predictions would be excluded manually.”
